# The Impact of Dating Applications on Adolescent Development: A Psychological Perspective

**DOI:** 10.3390/bs14030215

**Published:** 2024-03-06

**Authors:** Luca Cerniglia

**Affiliations:** Faculty of Psychology, International Telematic University, 00186 Roma, Italy; luca.cerniglia@uninettunouniversity.net

## 1. Introduction

The genesis of dating applications can be traced back to the early days of the internet, where websites served as the primary medium for digital dating. The transition from desktop websites to mobile apps has revolutionized the way individuals engage with these platforms, making them more accessible and integrated into daily life [1,2,3]. The introduction of smartphones equipped with GPS and other location-based services further catalyzed this shift, allowing apps like Tinder, launched in 2012, to popularize the concept of swiping for potential matches based on geographical proximity [4,5,6]. This evolution reflects broader technological and social trends, including increased internet penetration, the ubiquity of smartphones, and changing attitudes towards online dating. Dating apps have grown from a niche phenomenon to a mainstream method of seeking romantic and sexual partners, especially among younger demographics. The prevalence of these apps has been facilitated by their ease of use, the instant gratification they offer, and their ability to connect users with a vast network of potential partners [7,8,9].

Dating apps are characterized by their user-friendly interfaces, which often prioritize visual appeal. Features such as swiping in Tinder, location-based matching in Grindr, and the women-message-first approach in Bumble cater to different preferences and demographics, but all aim to streamline the process of finding a match [10,11,12]. These apps also incorporate various mechanisms to engage users, from gamification elements like swiping and matching to integrating social media profiles for authentication and personality insights. The design and functionality of these apps reflect and reinforce contemporary dating practices, emphasizing speed, efficiency, and convenience [13,14,15].

The use of dating apps is not uniform across all segments of the population. Factors such as age, gender, sexual orientation, and relationship status significantly influence app usage patterns. Young adults, particularly those belonging to sexual minorities, are more likely to use dating apps, seeking both long-term relationships and casual encounters [16].

The motivations behind using dating apps are multifaceted, ranging from seeking entertainment and casual sex to looking for serious relationships. This diversity in user intentions can lead to mismatches in expectations among app users, highlighting the complexity of digital dating dynamics [17,18,19,20].

Moreover, the use of dating apps has psychosocial implications, affecting users’ self-esteem, body image, and social relationships. The emphasis on physical appearance and the commodification of romantic interactions can exacerbate feelings of anxiety and inadequacy among users. However, these platforms also offer opportunities for positive social interactions, enabling users to explore their identities and connect with others who share similar interests and experiences [21].

## 2. Why Do Adolescents Use These Apps?

Adolescents utilize dating applications for a variety of reasons that extend beyond mere curiosity or the pursuit of romantic connections. A study by Sawyer, Smith, and Benotsch (2018) revealed that 39.5% of young heterosexual adults reported using dating apps, indicating a significant engagement among younger populations as well [22]. This engagement is not solely driven by the pursuit of romantic or sexual encounters but encompasses a broader spectrum of motivations including entertainment, exploration of identity, and socialization within peer groups. The digital landscape offers adolescents a platform for emotional expression and connection that is distinct from traditional social settings, providing a sense of safety and control over their social interactions [23,24].

Furthermore, the use of dating apps among adolescents is influenced by the developmental stage of exploring sexual and romantic identities. The anonymity and breadth of choice offered by these platforms allow for a less constrained exploration of these aspects of their identity compared to offline environments. This exploration is crucial during adolescence, a period characterized by significant developmental changes, impulsivity, psychopathological risk and the formation of sexual and romantic identities [25,26,27,28,29].

However, this engagement is not without its challenges. Adolescents navigating these digital platforms may encounter risks such as exposure to inappropriate content, cyberbullying, and the potential for exploitation. Despite these risks, the allure of dating apps for adolescents lies in their ability to meet developmental needs for autonomy, identity exploration, and social connection, underscoring the complex interplay between the benefits and challenges of digital dating platforms [30].

In summary, adolescents are drawn to dating apps for reasons that reflect their developmental stage, including the desire for entertainment, social connection, and exploration of sexual and romantic identities. While these platforms offer unique opportunities for development and exploration, they also present challenges that need to be navigated carefully.
